# Mapping Quantitative Trait Loci Associated With Graft (In)Compatibility in Apricot (*Prunus armeniaca* L.)

**DOI:** 10.3389/fpls.2021.622906

**Published:** 2021-02-19

**Authors:** Ana Pina, Patricia Irisarri, Pilar Errea, Tetyana Zhebentyayeva

**Affiliations:** ^1^Unidad de Hortofruticultura, Centro de Investigación y Tecnología Agroalimentaria de Aragón (CITA), Zaragoza, Spain; ^2^Instituto Agroalimentario de Aragón – IA2 (CITA-Universidad de Zaragoza), Zaragoza, Spain; ^3^The Schatz Center for Tree Molecular Genetics, Department of Ecosystem Science and Management, The Pennsylvania State University, University Park, PA, United States

**Keywords:** apricot, breeding, graft incompatibility, linkage map, quantitative trait loci, sequence-based genotyping

## Abstract

Graft incompatibility (GI) between the most popular *Prunus* rootstocks and apricot cultivars is one of the major problems for rootstock usage and improvement. Failure in producing long-leaving healthy grafts greatly affects the range of available *Prunus* rootstocks for apricot cultivation. Despite recent advances related to the molecular mechanisms of a graft-union formation between rootstock and scion, information on genetic control of this trait in woody plants is essentially missing because of a lack of hybrid crosses, segregating for the trait. In this study, we have employed the next-generation sequencing technology to generate the single-nucleotide polymorphism (SNP) markers and construct parental linkage maps for an apricot F_1_ population “Moniqui (Mo)” × “Paviot (Pa)” segregating for ability to form successful grafts with universal *Prunus* rootstock “Marianna 2624”. To localize genomic regions associated with this trait, we genotyped 138 individuals from the “Mo × Pa” cross and constructed medium-saturated genetic maps. The female “Mo” and male “Pa” maps were composed of 557 and 501 SNPs and organized in eight linkage groups that covered 780.2 and 690.4 cM of genetic distance, respectively. Parental maps were aligned to the *Prunus persica* v2.0 genome and revealed a high colinearity with the *Prunus* reference map. Two-year phenotypic data for characters associated with unsuccessful grafting such as necrotic line (NL), bark and wood discontinuities (BD and WD), and an overall estimate of graft (in)compatibility (GI) were collected for mapping quantitative trait loci (QTLs) on both parental maps. On the map of the graft-compatible parent “Pa”, two genomic regions on LG5 (44.9–60.8 cM) and LG8 (33.2–39.2 cM) were associated with graft (in)compatibility characters at different significance level, depending on phenotypic dataset. Of these, the LG8 QTL interval was most consistent between the years and supported by two significant and two putative QTLs. To our best knowledge, this is the first report on QTLs for graft (in)compatibility in woody plants. Results of this work will provide a valuable genomic resource for apricot breeding programs and facilitate future efforts focused on candidate genes discovery for graft (in)compatibility in apricot and other *Prunus* species.

## Introduction

The main goals of apricot breeding programs are to decrease production costs (pest and disease resistance), to increase yield (self-compatibility and graft compatibility, low or high chill requirement), and to improve fruit quality (Ruiz et al., 2010; Zhebentyayeva et al., 2012). Nowadays, improvements in cultural practices, the withdrawal of soil fumigants, extended cultivation season, and demand for higher fruit quality significantly increased the number of both rootstocks and apricot cultivars on the market. Graft compatibility between rootstock and scion becomes a major concern for advanced selections to be released for agricultural production. Knowledge on the extent and nature of (in)compatibility reaction provides growers and nurseries with information that allows to estimate weakness of the graft interface and potential risk of delayed incompatibility, i.e., long-term survival and functioning of the composite grafted plants (Hartmann et al., 2002; Pina et al., 2017; Baron et al., 2019). Early diagnostics of graft compatibility is of critical importance for plants with a long-life cycle. Therefore, characterization of the physiological and molecular mechanisms involved in graft responses at early stages of development was in focus of several studies in stone fruit trees (Irisarri et al., 2016; Reig et al., 2019) and grapevines (Cookson et al., 2014; Assunção et al., 2019). Differentially expressed transcripts, proteins, and secondary metabolites accumulated at the graft interface were reported in several studies to improve our understanding of the molecular-level differences between heterografts and homografts (Wang et al., 2016., Chen et al., 2017; Pina et al., 2017; Gautier et al., 2019; Prodhomme et al., 2019). However, the advancement of graft incompatibility (GI) studies was slow compared to other agronomic traits such as resistance to biotic and abiotic stresses, self-fertility, or fruit quality, because it is logistically challenging to graft hundreds of different scion–rootstock combinations with a sufficient number of replicates to quantify the trait. Additionally, accurate identification of expressed transcripts, proteins, or other biomolecules requires appropriate controls (ungrafted scions and rootstocks, homografts, and heterografts) and sufficient amount of sampled tissue (Goldschmidt, 2014; Gautier et al., 2019). Consequently, our knowledge on biology and genetics of rootstock–scion compatibility is incomplete and requires more studies. In the last decades, the development and application of molecular tools have increased the speed and precision of the breeding process in horticulture, particularly for traits that are difficult to evaluate phenotypically or when the expression of a gene is recessive. Molecular markers are frequently used for indirect selection on traits of interest in fruit trees and other crops (Arús et al., 2005; He et al., 2014). Because of long period of juvenility and space constrains, traditional selective breeding and marker-assisted selection are usually carried out in the *Prunus* breeding programs simultaneously. Selection by molecular markers is possible, provided sufficient mapping information is known in shortening the number of generations required to eliminate the undesired genes in the backcrossing programs (Ruiz et al., 2010; Aranzana et al., 2019). Thus, genetic linkage analysis of segregating progeny in biparental crosses elucidates structural organization of genomes and enables identification of genomic regions and their gene contents underlying simple Mendelian and complex quantitative traits. As a result, approximately 200 maps have been developed for more than 100 traits in the *Prunus* tree species (reviewed in Guajardo et al., 2015; Aranzana et al., 2019). The first apricot linkage maps were developed using the combination of different molecular markers (RAPDs, AFLPs, RFLPs, and SSRs) from different families that segregated for plum pox virus (PPV) resistance (Hurtado et al., 2002; Vilanova et al., 2003), bloom date (Campoy et al., 2011), self-incompatibility (Muñoz-Sanz et al., 2017), and fruit quality (Ruiz et al., 2010; Salazar et al., 2013; García-Gómez et al., 2019). Because of recent advancements in biotechnology, the use of single-nucleotide polymorphisms (SNPs) markers for genotyping has increased the potential to score variation in specific DNA targets. Sequence-based genotyping (SBG) with different modifications provides a rapid and low-cost approach to genotype breeding populations and their parents, allowing plant breeders to implement genetic linkage analysis, genome-wide association studies, and genomic selection (GS) under a large scale of plant breeding programs (Truong et al., 2012; Andrews et al., 2016; Scheben et al., 2017). It has been shown to be a valid tool for population genetics studies (Fu and Peterson, 2011; Peterson et al., 2014) and identification of quantitative trait loci (QTLs). In fruit trees, genotyping by sequencing to date was conducted in apple (Nocker and Gardiner, 2014), raspberry (Ward et al., 2013), cherry (Guajardo et al., 2015), peach (Bielenberg et al., 2015), Japanese plum (Salazar et al., 2017, 2019), and *Prunus* rootstock germplasm collections (Guajardo et al., 2020), generating SNPs of sufficient quality and quantity to be of utility in genetic mapping. In apricot, high-density genetic map was constructed and used for mapping pistil abortion, an important agronomic trait decreasing the yield in production (Zhang et al., 2019). However, the genetic control of GI remains poorly understood mainly because of a lack of hybrid crosses, segregating for the trait. To generate a hybrid plant material for genetic analysis, we cross-pollinated two apricot cultivars that were previously phenotyped as graft-compatible and -incompatible when grafted to the same rootstock universal for *Prunus*. Using this cross, we established a phenotyping protocol for this complex trait based on cytomorphological observations of graft interface (Irisarri et al., 2019). Regression analysis of phenotypic data across the progeny revealed likely polygenic control of successful graft formation. In the follow-up study presented here, we genotyped progeny by sequencing and constructed high-density parental genetic maps for QTL analysis in order to (1) answer a questions if any genomic regions in apricot are significantly associated with graft (in)compatibility trait and (2) delineate QTL intervals and identify genetic markers most associated with graft (in)compatibility trait for potential use in breeding.

## Materials and Methods

### Plant Material and DNA Extraction

We genotyped by sequencing a population of 138 F1 apricot individuals from the “Mo × Pa” cross between graft-incompatible traditional Spanish cultivar “Moniqui (Mo)” and graft-compatible French cultivar “Paviot (Pa)”. Genomic DNA was extracted from young leaves of each individual using the DNeasy plant kit (Qiagen, United States) according to the manufacturer’s instructions and quantified on agarose gels in presence of ethidium bromide against incremental dilutions of the lambda DNA standard (Green and Sambrook, 2012). As a first quality test, 100 ng of DNA from parents and some progeny individuals was digested with *Pst*1 and *Mse*1 (New England Biolabs, United Kingdom) using the manufacturer’s instructions. DNA samples were sent to the Clemson University Genomics institute (SC, United States) to perform SBG of offspring and parents.

### Data Processing and SNP Genotyping

DNA samples were digested with partially methylation-sensitive enzyme *Ape*K1, and 96-plexed libraries were prepared following the protocol described for maize by Elshire et al. (2011) with few modifications. Parental genotypes were sequenced three times and used as intraplate and interplate controls of sequencing quality. Data processing and SNP genotyping were done as previously described for multiple chestnut crosses (Zhebentyayeva et al., 2019). Reads were demultiplexed using “process_radtag” command implemented in the Stacks v.1.44 (Catchen et al., 2011). In total, 97% of reads were retained after check for quality (QC > 30) and presence of *Ape*K1 restriction site (Supplementary Table S1). Triplicated parental reads were combined, and names “p1” and “p2” for “Moniqui” and “Paviot” were given, respectively. Reads were then aligned against the *Prunus persica* v2.1 genome (Verde et al., 2017^1^) using short-read nucleotide alignment program GSNAP version 2015-07-23 at default parameters (Wu and Nacu, 2010). Also, Burrows–Wheeler aligner (BWA) by Li and Durbin (2009) was used to compare efficiency of two aligners for progeny genotyping.

Following the same strategy used for linkage mapping in heterozygous grape (Hyma et al., 2015), we first separated sequences into chromosomal groups based on alignment against the *P. persica* v 2.1 pseudochromosomes and collected genotypic data for linkage groups (pseudochromosomes) separately. Replicated parental reads were combined, providing saturated frameworks for SNP genotyping. A catalog of tags and SNP genotypes was generated using a “ref_map” command and encoded as an F_1_ segregating population type. Genotypes were further filtered for minimum stack depth of five (-m) and a minimum number of genotyped progenies at 90% necessary to retain any SNP locus (−*r*). Data were exported from Stacks in a JoinMap format and used for linkage map construction. Map graphics were generated with MapChart v. 3.0 (Voorrips, 2002).

### Linkage Map Construction

Two parental, female and male, maps for graft-incompatible “Moniqui” and graft-compatible “Paviot”, respectively, were constructed following the two-way pseudo-testcross strategy for outcrossing species (the CP population type) using the JoinMap v4.1 (Van Ooijen, 2006). An input file generated by Stacks was manually curated, and only markers polymorphic in one parent (< lm × ll > and < nn × np > configurations) were retained. Individuals with more than 10% of missing data as well as identical loci (>0.95 similarity threshold) were excluded from consideration. Additionally, distorted markers (*P* ≤ 0.05) were identified and deleted from dataset using the χ^2^ test and the “Exclude Selected Item” function in JoinMapv4.1. Phases (coupling and repulsion) of the marker loci were automatically detected with the CP option. Linkage groups were established at independence logarithm of odds (LOD) < 7.0 using the regression algorithm (Kosambi mapping function) with the following thresholds: recombination frequency of 0.400, LOD value of 1.0, and a goodness-of-fit jump of 5.00 and performing a ripple function after each added locus for optimization of marker order. Three rounds of mapping were performed. After an initial round of mapping, loci were excluded from subsequent maps if they (1) had a high nearest neighbor fit values and/or low locus mean genotype probabilities; (2) introduced negative genetic distance assigned to conflicting linkage phases; (3) produced multiple hits when blasted against the *P. persica* v2.1 genome; or (4) were in prominent order conflicts with *P. persica* v2.1 pseudochromosomes (few cases). Mapping iterations continued until there was no further improvement in map quality as assessed by χ^2^ values for each linkage group or alignment against peach reference genome. The Phytozome 12.1 genome browser (Goodstein et al., 2012) was used for verifying orientation of linkage groups and marker order along the *P. persica* v2.1 pseudochromosomes, as well as for functional annotation of marker sequences most associated with the trait.

### Analyzing QTLs Associated With Graft (In)Compatibility

**FIGURE 1:**
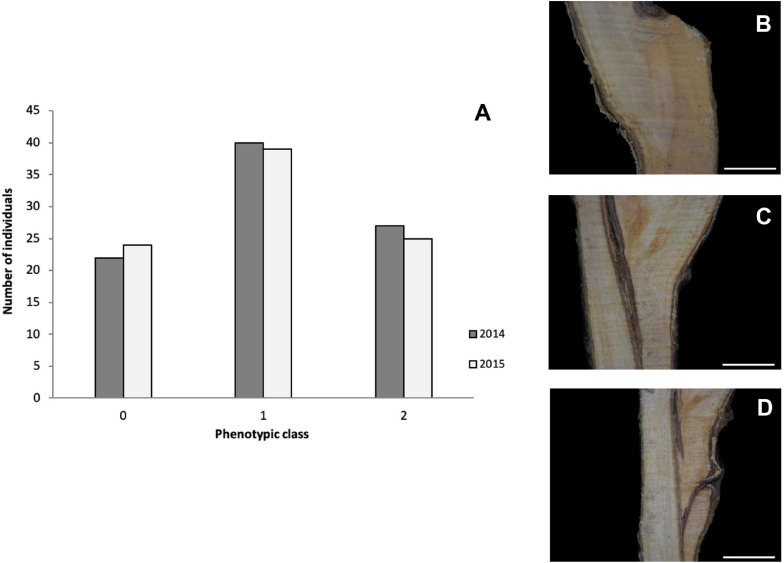
**(A)** Distribution of classified graft (in)compatibility phenotypic classes in the mapping population “Mo × Pa” progeny in the years 2014 and 2015. Images showing categories 0 **(B)**, 1 **(C)**, and 2 **(D)**. Scale bars = 5 mm. Category descriptions: (0) perfect union in which the graft line is almost invisible; (1) the bark and wood are continuous, although the line of union in the wood is often clearly distinguished by excessive ray formation; and (2) unions showing vascular and wood discontinuities.

Two-year phenotypic data, anatomical patterns associated with GI, collected for 92 individuals from the “Mo × Pa” cross in 2014 and 2015 (Irisarri et al., 2019) were used for detecting marker–trait associations in this study. Necrotic line (NL) and wood and bark discontinuities (WD and BD, respectively) were scored between 0 = absence and 5 = presence. For overall estimates of the trait, GI categories were assigned to individuals according to Herrero (1951) with some modifications: category 0 represents a perfect union in which the graft line is almost invisible; in category 1, the bark and wood are continuous, although the line of union in the wood is often clearly distinguished by excessive ray formation; and in category 2, unions show vascular discontinuities and WD (Figure 1). Five to 10 grafts on the rootstock “Marianna 2624” were phenotyped for each hybrid individual, and mode values were calculated for each parameter evaluated 1 year after grafting.

The QTL analyses were performed using multiple statistical analyses: non-parametric Kruskal–Wallis test (KW), interval (IM), and composite interval multiple (MQM) mapping implemented in the MapQTL6 (Van Ooijen, 2009). The minimum LOD score for QTL detection was determined by either the genome-wide or chromosome-wide LOD significance threshold (α = 0.05) calculated using 1,000 permutations (Churchill and Doerge, 1994). Marker–trait associations above chromosome-wide but below genome-wide thresholds were declared as suggestive QTLs following guidelines by Lander and Kruglyak (1995). The support intervals for QTLs were calculated using a 1.5-LOD drop interval. The QTL (q) names reflected the trait (NL, BD, WD, and GI) and their positioning on parental linkage groups (from LG1 trough LG8), and they were appended with a year of phenotyping. For example, the QTL named qGI.5-2014 was the first for GI on G5 from the 2014 cohort of the “Mo × Pa” cross.

## Results

### Sequence-Based Genotyping and Linkage Mapping

Altogether, 270,059,232 (97%) clean barcoded reads were generated for the “Mo × Pa” progeny. The average number of reads per individual was 1.93 mln (Supplementary Table S1). Seven individuals with less than 1-mln reads were discarded from genotyping. We compared the efficiency of the short-read aligners BWA and GSNAP to map apricot sequences against the reference *P. persica* v2.1 genome. Percent of unique reads properly aligned against reference with the GSNAP software was higher than that by BWA, 51.27 and 40.58%, respectively (data not shown). Consequently, the GSNAP dataset (individual bam files) was used for progeny genotyping. Catalog of tags, i.e., apricot DNA fragments potentially useful for SNP calling in progeny, was composed of 147,315 sequences. Altogether, 18,995 SNPs distributed along eight peach pseudochromosomes were written into unfiltered mapping files. Of these, 7,618 high-quality SNPs were present in more than 90% individuals (Table 1). These genotypes were exported from Stacks for additional filtering based on marker segregation types and χ^2^ goodness-of-fit test for distortion.

**TABLE 1:** Marker distribution in F1 “Moniqui × Paviot” cross (“Mo × Pa”).

Linkage group	Markers in configurations	Markers in configurations	Markers segregating in female parent	Markers segregating in male parent	Mo map			Pa map		
	<ab × cd >, <ef × e.g., >, <lm × ll >, <nn × np >, <hk × hk >	<lm × ll >, < nn × np >	<lm × ll >	<nn × np >	SNPs	Length (cM)	Density (cM per SNP)	SNPs	Length (cM)	Density (cM per SNP)
**G1**	1,835	544	293	251	91	124.5	0.229	80	160.7	2.01
**G2**	939	424	315	109	66	149.9	0.354	81	55.7	0.69
**G3**	764	268	219	49	74	61.9	0.231	23	52.0	2.26
**G4**	768	187	66	121	31	109.4	0.585	45	104.3	2.32
**G5**	825	396	180	216	111	53.6	0.135	49	93.3	1.90
**G6**	1,100	551	207	344	59	57.0	0.103	104	51.2	0.49
**G7**	622	222	101	121	39	103.4	0.466	36	118.1	3.28
**G8**	765	308	170	138	86	120.6	0.392	83	55.1	0.66
**Total**	7,618	2,900	1,551	1,349	557	780.3	0.269	501	690.4	1.38

*lm × ll, SNPs segregating only in “Moniqui”; nnxnp, SNPs segregating only in “Paviot”; hkxhk, SNPs segregating in both parents.*

**FIGURE 2:**
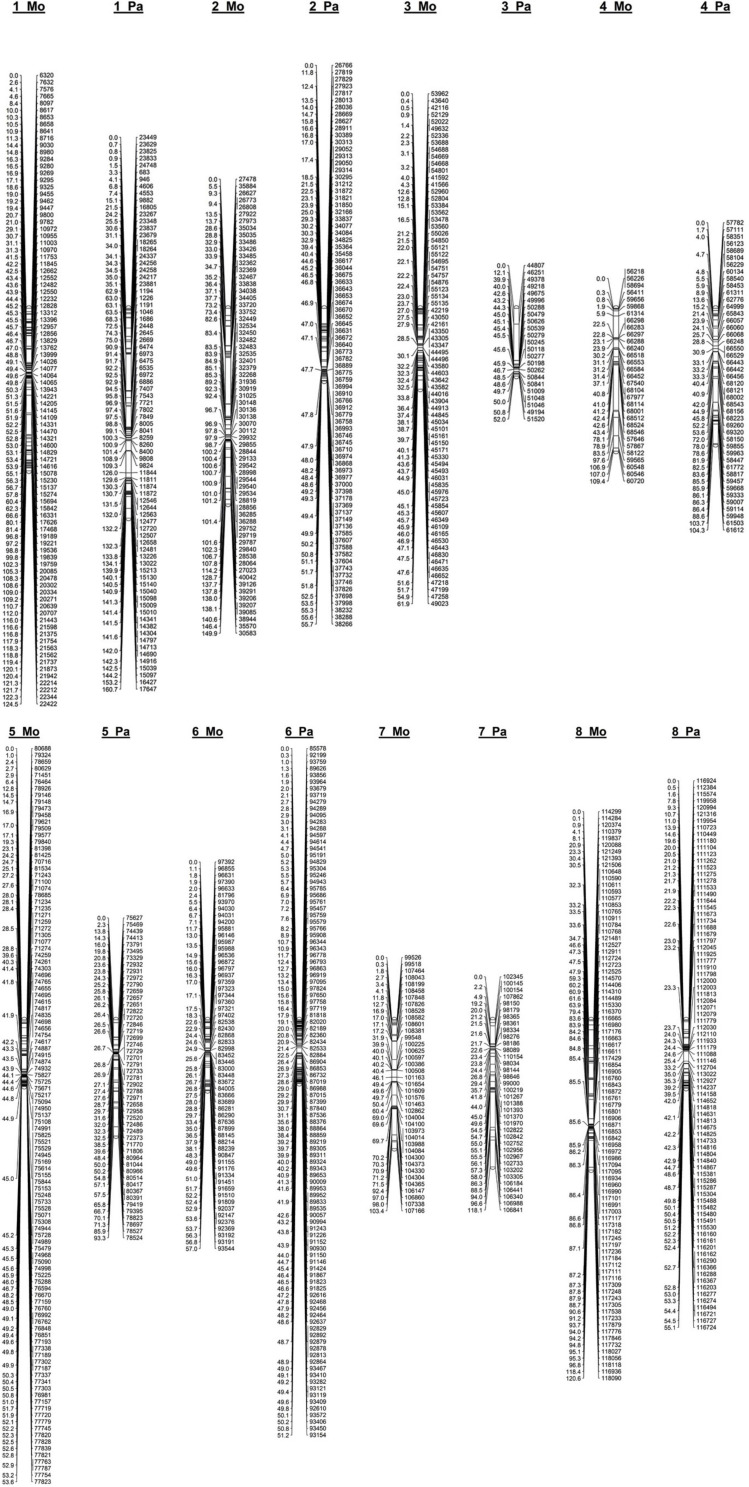
Linkage map constructed from genotyping-by-sequencing derived SNPs in an F1 apricot population derived from a cross between the female parent “Moniqui” and the male parent “Paviot” (*N* = 138 seedlings).

After filtering markers heterozygous in one parent (segregation types either < lm × ll > or < nn × np >), the number of markers was dramatically reduced. Altogether, 1,551 (23%) and 1,349 (18%) SNPs distributed along eight *P. persica* pseudochromosomes satisfied a marker configuration requirement in female and male parents, respectively. These were assessed for deviation from segregation ratio 1:1 using the χ^2^ goodness-of-fit tests (at *P* ≤ 0.05). Finally, 577 and 501 non-distorted SNPs were organized in eight female “Mo” and male “Pa” linkage groups, respectively (Table 1 and Figure 2). The resultant genetic maps spanned a total length of 780.3 and 690.4 cM, respectively. The LG length was variable in the female “Mo” map, with LG2 being the largest, 149.9 cM, and LG5 the shortest, 53.3 cM group. The average marker density was 1.4 cM per marker. The male “Pa” map was composed of maximum and minimum linkage group lengths of 160.7 cM (LG1) and 51.2 cM (LG6) and an average marker density of 1.38 cM per marker (Figure 1). The average distance between markers ranged from 0.48 cM (LG5) to 3.53 cM (LG4) in the “Mo” and from 0.49 (LG6) to 3.28 (LG7) in the “Pa” maps. The markers mapped to the two parental maps, along with the linkage group, segregation type, phase, and the “Mo × Pa” encoded alleles, are detailed in Supplementary Table S2.

We compared marker order on the genetic maps calculated from recombination frequencies and physical order of corresponding sequencing tags on the assembled *P. persica* pseudochromosomes (Supplementary Table S3). The parental “Mo × Pa” maps were highly syntenic, and the SNP marker order was in good agreement with physical positioning markers in the *P. persica* genome. However, several genomic regions of genetic maps were underrepresented in consequence of removal of the SNP markers with segregation ratios deviating from 1:1. Several gaps larger than 5 Mb of physical distance were identified—one on the top of the LG1 on the “Mo” map (0–23 Mb) and three on the LG1 (39.2–47.9 Mb), LG2 (1.2–14.9 Mb), and LG3 (0–18.2 Mb) of the “Pa” map. As a result, corresponding linkage groups, the LG1 on female “Mo” map, and LG1, LG2, and LG3 of the male “Pa” map, were significantly shorter than reference *Prunus* map. Similar non-random effect of segregation distortion on parental maps was reported for *Rubus idaeus* (Ward et al., 2013).

### QTL Analysis for GI Traits in Apricot

Altogether, 92 seedlings from the “Mo × Pa” progeny, phenotyped in the years 2014 and 2015 for graft (in)compatibility (GI), and three phenotypic characters linked to this trait NL, BD, and WD were evaluated (Irisarri et al., 2019). For reasons not related to phenotyping, five grafted trees died in 2014 (no phenotypic data recorded), and one tree died in 2015 (2014 phenotypic data only). Phenotypic data for specific characters associated with graft (in)compatibility and overall GI scores were used for mapping QTLs on the “Mo” and “Pa” maps (Supplementary Table S4). QTLs were mapped separately on the “Mo” and “Pa” maps because cultivars could contribute to graft compatibility using different set of genes, not necessarily colocalized on same linkage groups. Mapped markers associated with graft (in)compatibility characters in female parent (the “Mo” map) were not detected. Apparently, inheritance of the trait by progeny relied solely on male parent “Paviot” graft-compatible with “Marianna 2624.” Therefore, herein we report the QTL analysis results generated with the male “Pa” map. Using three statistical methods (KW, IM, and MQM) implemented in MapQTL, we identified two genomic regions on LG5 (44.9–60.8 cM) and LG8 (33.2–39.2 cM) associated with graft (in)compatibility characters such as NL, WD, and overall GI scores (Table 2). However, we failed to detect QTL signals, i.e., marker–trait associations, for BD in both 2014 and 2015 datasets. Two QTLs qWD8-2014 and qNL8-2015 explaining 16.1 and 14.7% of phenotypic variance colocalized on LG8 and shared the same genetic interval with a qWD8-2015 and qGI8-2015. However, the last two suggestive QTLs were significant only at a chromosome-wide LOD threshold. On LG5, two colocalized putative QTLs qNL5-2014 and qGI-2015 were also significant at chromosome-wide level. The KW test provided additional support for positioning graft compatibility QTLs on the “Pa” genetic map for those datasets, in which QTLs were not declared because of low (<1.0) LOD scores. Most significant markers pa71770 and pa71806 on LG5 for the qWD5-2014, qGI5-2015, and pa113022 for the qGI8-2014 on LG8 were shared among all graft compatibility traits. Thus, two genomic regions on LG5 and LG8 were associated with graft compatibility, although at different significance levels, depending on specific character and year (Supplementary Figure S1).

**TABLE 2:** Quantitative trait loci (QTLs) associated with necrotic line (NL), wood (WD) and bark discontinuity (BD), and overall graft incompatibility (GI) on the male “Paviot” map.

Year	QTL	LG	Confactor	Confidence interval		Physical position		LOD	Phenotypic	Kruskal–Wallis test	
			Marker	Start (cM)	End (cM)	Start (bp)	End (bp)		variance (%)	*K*	Significance
**2014**	**qNL5-2014**	5	**pa71770**	44.9	60.8	9,141,408	12,368,644	**1**.**91**	9.7	9,515	***
	qWD5-2014	5	pa71770	–	–	–	–	1.03 (ns*)	–	5,344	**
	**qWD8-2014**	**8**	**pa113022**	33.2	39.2	15,360,275	17,468,552	**3**.**28**	16.1	16,179	*******
	**qGI5-2014**	**5**	**pa71806**	44.9	60.8	9,141,408	12,368,644	**2**.**12**	10.7	9,402	****
	qGI8-2014	8	pa113022	–	–	–	–	1.31 (ns*)	–	4,936	**
**2015**	**qNL8-2015**	**8**	**pa113022**	33.2	39.2	15,360,275	17,468,552	**3**.**01**	14.7	4,880	**
	**qWD8-2015**	**8**	**pa113022**	33.2	39.2	15,360,275	17,468,552	**2**.**23**	11.4	6,874	***
	qGI5-2015	5	pa71806	–	–	–	–	1.41 (ns*)	–	6,089	**
	**qGI8-2015**	**8**	**pa113022**	33.2	39.2	15,360,275	17,468,552	**1**.**99**	10.1	6,549	**

*Not significant at chromosome-wide LOD threshold. The Kruskal–Wallis test significance: **0.01, ***0.001, ****0.0001, and *******0.0000001. Bold values provide QTLs significant at chromosome wide level

Using marker sequence information, we delineated QTL intervals for graft compatibility on the *P. persica* v2.1 pseudochromosomes (Table 2). The QTL intervals covered 3.2 Mb of physical distance on LG5 (9.14–12.36 Mb) and 2.1 Mb on LG8 (15.36–17.47 Mb). On LG5, two cofactors, i.e., most associated markers, pa71770 or pa71806, were located approximately 34 kb apart within the same QTL interval. The pp71770 derived from Prupe.5G111500.1 gene encoding a dirigent-like protein. Dirigent proteins were found to mediate a stereoselective bimolecular phenoxy radical coupling during lignan biosynthesis and modulate cell wall metabolism under stress condition (Paniagua et al., 2017). The pa71806 marker is derived from two overlapping genes of unknown functions, Prupe.5G111900.1 (+) and Prupe.5G112000.1 (-) encoding glyoxalase-like domain protein and acyl-coenzyme A:6-aminopenicillanic acid acyltransferase, respectively. The most associated marker pa113022 identified within QTL interval on LG8 is derived from gene Prupe.8G141300.1 annotated as amino acid permease family protein involved into transport amino acids into cell.

## Discussion

Graft incompatibility is an important agronomical trait in the development and selection of new rootstocks and cultivars. Although several approaches have been applied to identify physiological and molecular markers related to graft union formation and graft success, there is still limited information on this trait. Several studies have monitored the transcripts and proteins associated with graft union formation and GI in grape (Cookson et al., 2013, 2014), pecan (Mo et al., 2018), and citrus (He et al., 2018). Higher expression of genes related to stresses, wounding, and secondary metabolism has been highlighted as typical for less compatible scion–rootstock combinations at early stages of development after grafting: in lichi (Chen et al., 2017), grapevine (Cookson et al., 2014; Assunção et al., 2019), *Prunus* species (Irisarri et al., 2015, 2016), melon (Aloni et al., 2008), and bottle gourd (Wang et al., 2016). However, not all the studies included all the necessary controls to reliably identify the genes and proteins differentially expressed during heterografting and associated with GI. Similarly, the metabolite profile of the scion, rootstock, and graft interfaces appears to change over time, suggesting that metabolite markers of GI could be specific and only valid in understanding graft (in)compatibility at certain developmental stages (Assunção et al., 2019). Until now, no genetic approaches have been used due to different reasons: the challenge of phenotyping GI in large fruit tree population, which is extremely labor-intensive and requires space for growing trees, and the difficulty of finding phenotypically different parents in respect of graft compatibility but missing fertilization barriers. So far, apricot was the only fruit tree species for which an F_1_ cross segregating for graft compatibility was generated. A phenotyping protocol was established and used to demonstrate that progeny segregate for the trait (Irisarri et al., 2019).

In this report, we utilized the SBG technology to generate extensive dataset of the SNP markers distributed throughout the apricot (*Prunus armeniaca*) genome. Parental maps constructed for the “Mo × Pa” cross provided sufficient marker density for downstream QTL analyses and were comparable with recently released saturated genetic maps for apricot cross segregating for pistil abortion trait (Zhang et al., 2019). Similar marker density was reported in other *Prunus* species such as peach (Bielenberg et al., 2015), cherry (Klagges et al., 2013; Calle et al., 2018), and Japanese plum (Salazar et al., 2017). As indicated by marker order extrapolated to the *P. persica* pseudochromosomes (Supplementary Table S3), our apricot maps showed high colinearity with other *Prunus* maps and provided robust framework for QTL detection. Because of highly syntenic genomes and transferability markers across the *Prunus* maps (Verde et al., 2013), results of our study could be transferred to other species. Thus, the linkage maps constructed in this work provide a valuable genomic resource for apricot breeding programs and present an important tool for finding candidate genes underlying traits of interest for effective marker-assisted breeding.

In previous publication, anatomical and cytomorphological characteristics related to graft (in)compatibility displayed continuous variation within the progeny, suggesting a polygenic inheritance that implies interactions of a number of small-effect QTLs (Irisarri et al., 2019). In this study, we identified only two genomic regions on LG5 and LG8 associated with graft compatibility in progeny inherited from male parent “Pa”. The QTL interval on LG8 covering 3-cM interval on genetic map was supported by two QTLs the qNL8-2015 and qWD8-2014 and two suggestive QTLs, the qWD8-2015 and qGI8-2015. Thus, the LG8 QTL was more consistent across different years and traits reflecting specific aspects of graft-compatibility phenotype in progeny, whereas the QTL interval on LG5 was supported by two suggestive 1-year QTLs qNL5-2014 and qGI-2014. Putative QTLs declared at a chromosome-wide LOD thresholds are rarely reported in plant genetic studies. However, in animal genetics dealing with low progeny sizes and complex physiological traits or disease incidence, suggestive QTLs allowed initial delineation of genomic intervals associated with a number of traits in mice (Makhanova et al., 2017; Suto and Kojima, 2019), chicken (Besnier et al., 2011), and carp (Lv et al., 2016). In *Prunus*, there are several important agronomical traits that were mapped with low progeny sizes and candidate genes identified, for example, PPV resistance and self-incompatibility in apricot (Vilanova et al., 2003; Zuriaga et al., 2013; Muñoz-Sanz et al., 2017), adaptability to chilling in peach (Bielenberg et al., 2015), or fruit quality (Abdelghafar et al., 2020). In this study, we reported suggestive QTLs following a similar conservative approach that allows keeping maximum information on potential QTLs for their consequent verification with increased population size, improved phenotyping protocols, or application of high-throughput sequencing technologies for transcriptome analyses.

Formation of graft–scion union is a complex physiological process that involves multilayered regulations for establishing cell contact at graft interface and maintenance of nutrient and water balance between scion and rootstock (Pina et al., 2017). Connectivity in bark and xylem can be spatially or timely desynchronized during graft union formation. Distribution of the BD phenotypic data in the “Mo × Pa” progeny (Irisarri et al., 2019) was in agreement with the observation by Reig et al. (2019) that BD observed after grafting may be healed in older trees not leaving visible signs of disconnection. Thus, the QTL signals not detected with phenotypic BD dataset could be explained by problem with phenotyping or indicate involvement of additional genetic factors not accounted for in our study. The process of graft union formation begins with the formation of a necrotic layer, followed by the adhesion of both graft partners, callus cell formation, vascular cambium formation from the callus bridge, and the establishment of functional vascular connections (new xylem and phloem) between the stock and scion (Pina et al., 2017; Gautier et al., 2019). In most situations, profuse callusing causes the necrotic layer to disappear, but sometimes the persistence of the NL seems to inhibit the vascular differentiation producing unsuccessful graft combinations (Hartmann et al., 2002). Based on histological data and anatomy of the graft interface, graft incompatible combinations exhibit vascular discontinuities that are associated with presence of necrotic cells in the wood and bark, the inclusion of unlignified cells in the wood, and invaginations or breaks in the cambium (Ermel et al., 1999; Pina et al., 2017). Probably, the sequential events during graft union formation are not synchronized in a tissue-specific manner and were dependent on stage of development, rootstock–scion age, and maturity of grafts. This may also explain a year variation in intensity of the QTL signals on LG5 and LG8 for NL, WD, and GI reported here. In grape, graft success in heterografts also varied from year to year and was dependent on genetic background of scions (Assunção et al., 2019). Environmental effect on rootstock–scion interaction was studied mainly in vegetables that have less stringent labor and space limitations conducting experiments compared with perennial trees (Albacete et al., 2015; Djidonou et al., 2020). Environmental influences have an effect on the anatomical structure, as well as on the physical and chemical properties of wood formation including the cambium, phloem, and bark (Battipaglia et al., 2014).

Delineated QTL intervals on LG5 and LG8 cover 3.2 and 2.1 Mb of physical distance on *Prunus* pseudochromosomes 5 and 8, respectively. Hundreds of genes are annotated in genomic regions underlying reported QTLs. Results of our analyses highlighted markers pa71770 on LG5 and pa113022 on LG8 derived from expressed *Prunu*s genes as most associated with QTLs. The first one, the Prupe.5G111500.1 encoding a dirigent-like protein, could be potentially involved in lignin biosynthesis and cell wall formation (Paniagua et al., 2017). Increased phenolic production in graft interface is reported to be the earliest manifestation of failure to form a union between rootstock and scion in *Prunus* species (Usenik et al., 2006; Pina et al., 2017), grape (Canas et al., 2015; Assunção et al., 2016), and pear (Musacchi et al., 2000; Hudina et al., 2014). Expression of phenylalanine ammonia lyase, a key enzyme in the synthesis of phenolic compounds, has a prominent effect on their accumulation in incompatibility response (Irisarri et al., 2016). The second gene highlighted in our study on LG8, Prupe.8G141300.1, was annotated as membrane permeases involved in the transport of amino acids into the cell (Fujita and Shinozaki, 2014). In peach/plum grafts, a decrease in free amino acids was shown in incompatible grafts, while they became stabilized in compatible grafts between 79 and 89 days after grafting (Moreno et al., 1994). However, it is premature to consider genes underlying most associated markers within QTL intervals as candidate genes for graft-incompatibility trait. Answering this question requires verification of QTLs and marker–trait associations in other than “Mo × Pa” genetic background and extensive transcriptome studies in apricot graft-compatible and -incompatible interface to identify differentially expressed genes within QTL intervals. It is likely that different systematic groups of plants share common molecular network involved in graft union formation. Significant differentially expressed genes were identified and analyzed between compatible and incompatible combinations involved in metabolism (carbohydrate, energy), wound response, phenylpropanoid biosynthesis, and plant hormone signal transduction in Litchi (Chen et al., 2017), citrus (He et al., 2018), and grape (Assunção et al., 2019). More studies are necessary including the appropriate controls (homografts, ungrafted and wounded rootstock, and scion tissues) to use transcriptome datasets generated for successful and failed heterografts in woody plants for candidate gene discovery in apricot. Thus, results of QTL mapping reported here could provide directions to more comprehensive and focused experiments on graft (in)compatibility trait. It would be valuable to study if any genes within QTL intervals are differentially expressed in graft combinations with different degree of compatibility. In addition, further analysis of non-related breeding material is necessary to validate the presence of putative QTLs. Increasing a progeny size of the “Mo × Pa” cross as well as additional years of observation may lead to the identification of additional QTLs for more complete characterization of genetic architecture of graft (in)compatibility in apricot. Therefore, more experimental strategies that lead to segregating families for graft (in)compatibility are crucial for further genetic characterization of this agronomic trait.

## Conclusion

This work highlighted that SBG is a rapid and suitable method for genetic map construction in an F_1_ apricot progeny segregating for the graft (in)compatibility trait. Our findings presented here provide a set of sequence-based SNPs useful for screening in apricot breeding programs. Furthermore, we constructed parental genetic maps and delineated genomic regions associated with graft (in)-compatibility parameters linked with the trait (NL, BD, and WD). QTLs with a significant effect through the years were found in LG8 as well as suggestive QTLs on LG5. Validation of these QTLs in other apricot progenies will help to set up marker-assisted breeding for this important trait in apricot. Likewise, the genetic information reported here can serve as the starting point for downstream genetic investigations such as QTL analyses, positional cloning of genes controlling traits of interest, and the development of GS strategies. The results presented in this article (map construction and QTLs found) should facilitate future work focused on exploring and understanding the genetic control of GI in *Prunus* species, as well as for searching candidate genes linked to this trait.

## Data Availability Statement

The datasets presented in this study can be found in online repositories. The names of the repository/repositories and accession number(s) can be found below: SRA, PRJNA675136.

## Author Contributions

AP and TZ designed and coordinated the research. AP and PI conducted the experiments and analysed and interpreted the data. PE contributed with experimental design. TZ carried out linkage mapping and QTL analysis. AP and TZ wrote the manuscript. All authors read, revised, and approved the manuscript.

## Conflict of Interest

The authors declare that the research was conducted in the absence of any commercial or financial relationships that could be construed as a potential conflict of interest.

## Footnotes

1 https://www.rosaceae.org/species/prunus_persica/genome_v2.0.a1

## Supplementary Material

Supplementary Figure 1 Colocalization of quantitative trait loci (QTLs) for graft (in)compatibility traits in the “Mo × Pa” cross. QTLs for necrotic line (qNL), wood discontinuity (qWD), and overall graft (in)compatibility (qGI) detected in 2014 and 2015 were drawn as blue, black, and red bars, respectively, along linkage groups with MapChart 3.0 (Voorrips, 2002). The most significant markers associated with QTLs and their positioning on maps are shown.

Supplementary Table 1 Statistics of sequencing data processing for parental and progeny genotypes in the “Mo × Pa” cross.

Supplementary Table 2 Encoded genotypes in JoinMap format used in constructing female “Moniqui” and male “Paviot” maps.

Supplementary Table 3 Alignment of female “Moniqui” and male “Paviot” genetic maps against the *P. persica* v2.0 pseudochromosomes.

Supplementary Table 4 Graft (in)compatibility traits phenotyped in the “Mo × Pa” progeny and parental genotypes.

## References

[B1] Abdelghafar A. da Silva Linge C. Okie W. R. Gasic K. (2020). Mapping QTLs for phytochemical compounds and fruit quality in peach. *Mol Breed.* 40:32. 10.1007/s11032-020-01114-y

[B2] Albacete A. Martínez-Andújar C. Martínez-Pérez A. Thompson A. J. Dodd I. C. Pérez-Alfocea F. (2015). Unravelling rootstock×scion interactions to improve food security. *J. Exp. Bot.* 66 2211–2226. 10.1093/jxb/erv027 25754404 PMC4986720

[B3] Aloni B. Karni L. Deventurero G. Levin Z. Cohen R. Katzir N. (2008). Physiological and biochemical changes at the rootstock-scion interface in graft combinations between cucurbita rootstocks and melon scion. *J. Hortic. Sci. Biotech.* 83 777–783.

[B4] Andrews K. R. Good J. M. Miller M. R. Luikart G. Hohenlohe P. A. (2016). Harnessing the power of RADseq for ecological and evolutionary genomics. *Nat. Rev. Genet.* 17 81–92. 10.1038/nrg.2015.28 26729255 PMC4823021

[B5] Aranzana M. J. Decroocq V. Dirlewanger E. Eduardo I. Gao Z. S. Gasic K. (2019). *Prunus* genetics and applications after de novo genome sequencing: achievements and prospects. *Hortic. Res.* 6:58. 10.1038/s41438-019-0140-8 30962943 PMC6450939

[B6] Arús P. Howad W. Mnejja M. (2005). “Marker development and marker-assisted selection in temperate fruit trees,” in *In the Wake of the Double Helix: From the Green Revolution to the Gene Revolution*, eds Tuberosa R. Phillips R. L. Gale M. (Bologna: Avenue media), 309–325.

[B7] Assunção M. Canas S. Cruz S. Brazão J. Zanol G. Eiras-Dias J. E. (2016). Graft compatibility of *Vitis* spp.: the role of phenolic acids and flavanols. *Sci. Hortic.* 207 140–145. 10.1016/j.scienta.2016.05.020

[B8] Assunção M. Santos C. Brazão J. Eiras-Dias J. E. Fevereiro P. (2019). Understanding the molecular mechanisms underlying graft success in grapevine. *BMC Plant. Biol.* 19:396. 10.1186/s12870-019-1967-8 31510937 PMC6737599

[B9] Baron D. Esteves Amaro A. C. Pina A. Ferreira G. (2019). An overview of grafting re-establishment in woody fruit species. *Sci. Hortic.* 243 84–91. 10.1016/j.scienta.2018.08.012

[B10] Battipaglia G. De Micco V. Sass-Klaassen U. Tognetti R. Mäkelä A. (2014). Wood growth under environmental changes: the need for a multidisciplinary approach. *Tree. Physiol.* 34 787–791. 10.1093/treephys/tpu076 25187625

[B11] Besnier F. Wahlberg P. Rönnegård L. Weronica E. K. Andersson L. Siegel P. B. (2011). Fine mapping and replication of QTL in outbred chicken advanced intercross lines. *Genet Sel. Evol.* 43:3. 10.1186/1297-9686-43-3 21241486 PMC3034666

[B12] Bielenberg D. G. Rauh B. Fan S. Gasic K. Abbott A. G. Reighard G. L. (2015). Genotyping by sequencing for SNP-based linkage map construction and QTL analysis of chilling requirement and bloom date in peach [*Prunus persica* (L.) Batsch]. *PLoS One* 10:e0139406. 10.1371/journal.pone.0139406 26430886 PMC4592218

[B13] Calle A. Cai L. Iezzoni A. Wünsch A. (2018). High-density linkage maps constructed in sweet cherry (*Prunus avium* L.) using cross- and self-pollination populations reveal chromosomal homozygosity in inbred families and non-syntenic regions with the peach genome. *Tree Genet. Genom.* 14:37. 10.1007/s11295-018-1252-2

[B14] Campoy J. A. Ruiz D. Egea J. Rees D. J. G. Celton J. M. Martínez-Gómez P. (2011). Inheritance of flowering time in apricot (*Prunus armeniaca* L.) and analysis of linked quantitative trait loci (QTLs) using simple sequence repeat (SSR) markers. *Plant. Mol. Biol. Report.* 29 404–410. 10.1007/s11105-010-0242-9

[B15] Canas S. Assunção M. Brazão J. Zanol G. Eiras-Dias J. E. (2015). Phenolic compounds involved in grafting incompatibility of *Vitis* spp: development and validation of an analytical method for their quantification. *Phytochem. Anal.* 26 1–7. 10.1002/pca.2526 24888592

[B16] Catchen J. Amores A. Hohenlohe P. Cresko W. Postlethwait J. (2011). Stacks: building and genotyping loci de novo from short-read sequences. *G3 Genes Genom. Genet.* 1 171–182. 10.1534/g3.111.000240 22384329 PMC3276136

[B17] Chen Z. Zhao J. Hu F. Qin Y. Wang X. Hu G. (2017). Transcriptome changes between compatible and incompatible graft combination of *Litchi chinensis* by digital gene expression profile. *Sci. Rep.* 7 1–12. 10.1038/s41598-017-04328-x 28638079 PMC5479835

[B18] Churchill G. A. Doerge R. W. (1994). Empirical threshold values for quantitative trait mapping. *Genetics* 138 963–971. 7851788 10.1093/genetics/138.3.963 PMC1206241

[B19] Cookson S. J. Clemente Moreno M. J. Hevin C. Nyamba Mendome L. Z. Delrot S. Magnin N. (2014). Heterografting with nonself rootstocks induces genes involved in stress responses at the graft interface when compared with autografted controls. *J. Exp. Bot.* 65 2473–2481. 10.1093/jxb/eru145 24692649 PMC4036518

[B20] Cookson S. J. Clemente Moreno M. J. Hevin C. Nyamba Mendome L. Z. Delrot S. Trossat-Magnin C. (2013). Graft union formation in grapevine induces transcriptional changes related to cell wall modification, wounding, hormone signalling, and secondary metabolism. *J. Exp. Bot.* 64 2997–3008. 10.1093/jxb/ert144 23698628 PMC3741690

[B21] Djidonou D. Leskovar D. I. Joshi M. Jifon J. Avila C. A. Masabni J. (2020). Stability of yield and its components in grafted tomato tested across multiple environments in Texas. *Sci. Rep.* 11:10. 10.1038/s41598-020-70548-3 32782333 PMC7419296

[B22] Elshire R. J. Glaubitz J. C. Sun Q. Poland J. A. Kawamoto K. Buckler E. S. (2011). A robust, simple genotyping-by-sequencing (GBS) approach for high diversity species. *PLoS One* 6:e019379. 10.1371/journal.pone.0019379 21573248 PMC3087801

[B23] Ermel F. F. Kervella J. Catesson A. M. Poëssel J. L. (1999). Localized graft incompatibility in pear/quince (*Pyrus communis*/*Cydonia oblonga*) combinations: multivariate analysis of histological data from 5-month-old grafts. *Tree Physiol.* 19 645–654. 10.1093/treephys/19.10.645 12651320

[B24] Fu Y.-B. Peterson G. W. (2011). Genetic diversity analysis with 454 pyrosequencing and genomic reduction confirmed the eastern and western division in the cultivated barley gene pool. *Plant. Genome* 4 226–237. 10.3835/plantgenome2011.08.0022

[B25] Fujita M. Shinozaki M. (2014). Identification of polyamine transporters in plants: paraquat transport provides crucial clues, plant. *Cell. Physiol.* 55 855–861. 10.1093/pcp/pcu032 24590488

[B26] García-Gómez B. Salazar J. Dondini L. Martinez-Gomez P. Ruiz D. (2019). Identification of QTLs linked to fruit quality traits in apricot (*Prunus armeniaca* L.) and biological validation through gene expression analysis using qPCR. *Mol. Breed.* 39:28. 10.1007/s11032-018-0926-7

[B27] Gautier A. T. Chambaud C. Brocard L. Ollat N. Gambetta G. A. Delrot S. (2019). Merging genotypes: graft union formation and scion-rootstock interactions. *J. Exp. Bot.* 70 805–815. 10.1093/jxb/ery422 30481315

[B28] Goldschmidt E. E. (2014). Plant grafting: new mechanisms, evolutionary implications. *Front. Plant. Sci.* 5:727. 10.3389/fpls.2014.00727 25566298 PMC4269114

[B29] Goodstein D. M. Shu S. Howson R. Neupane R. Hayes R. D. Fazo J. (2012). Phytozome: a comparative platform for green plant genomics. *Nucleic Acids Res.* 40 D1178–D1186. 22110026 10.1093/nar/gkr944 PMC3245001

[B30] Green M. R. Sambrook J. (2012). *Molecular Cloning: A Laboratory Manual (Fourth Revised Edition).* Cold Spring Harbor, NY: Cold Spring Harbor Laboratory Press.

[B31] Guajardo V. Solís S. Almada R. Saski C. Gasic K. Moreno M. A. (2020). Genome-wide SNP identification in *Prunus* rootstocks germplasm collections using genotyping-by-sequencing: phylogenetic analysis, distribution of SNPs and prediction of their effect on gene function. *Sci. Rep*. 10:1467. 10.1038/s41598-020-58271-5 32001784 PMC6992769

[B32] Guajardo V. Solís S. Sagredo B. Gainza F. Muñoz C. Gasic K. (2015). Construction of high density sweet cherry (*Prunus avium* L.) linkage maps using microsatellite markers and SNPs detected by genotyping-by-sequencing (GBS). *PLoS One* 10:e0127750. 10.1371/journal.pone.0127750 26011256 PMC4444190

[B33] Hartmann H. T. Kester D. E. Davies F. T. Geneve R. L. (2002). “Principles of grafting and budding,” in *Plant Propagation. Principles and Practices*, ed. Education P. (New York, NY: Prentice hall), 411–460.

[B34] He J. Zhao X. Laroche A. Lu Z. Liu H. Li Z. (2014). Genotyping-by-sequencing (GBS), an ultimate marker-assisted selection (MAS) tool to accelerate plant breeding. *Front. Plant. Sci.* 5:484. 10.3389/fpls.2014.00484 25324846 PMC4179701

[B35] He W. Wang Y. Chen Q. Sun B. Tang H. R. Pan D. M. (2018). Dissection of the mechanism for compatible and incompatible graft combinations of *Citrus grandis* (L.) Osbeck (‘Hongmian Miyou’). *Int. J. Mol. Sci.* 8:19. 10.3390/ijms19020505 29419732 PMC5855727

[B36] Herrero J. (1951). Studies of compatible and incompatible graft combinations with special reference to hardy fruti trees. *J. Hort. Sci.* 26 186–237.

[B37] Hudina A. Orazen M. Jakopic P. J. Stampar F. (2014). The phenolic content and their involvement in the graft incompatibility process of various pear rootstocks (*Pyrus communis* L.). *J. Plant Physiol.* 171 76–84. 10.1016/j.jplph.2013.10.022 24484960

[B38] Hurtado M. Romero C. Vilanova S. Abbott G. Llácer G. Badenes L. (2002). Genetic linkage maps of two apricot cultivars (*Prunus armeniaca* L.), and mapping of PPV (sharka) resistance. *Theor. Appl. Genet.* 105 182–191. 10.1007/s00122-002-0936-y 12582518

[B39] Hyma K. E. Barba P. Wang M. Londo J. P. Acharya C. B. Mitchell S. E. (2015). Heterozygous mapping strategy (HetMappS) for high resolution genotyping-by-sequencing markers: a case study in grapevine. *PLoS One* 10:e0134880. 10.1371/journal.pone.0134880 26244767 PMC4526651

[B40] Irisarri P. Binczycki P. Errea P. Martens H. J. Pina A. (2015). Oxidative stress associated with rootstock-scion interactions in pear/quince combinations during early stages of graft development. *J. Plant Physiol.* 176 25–35. 10.1016/j.jplph.2014.10.015 25543953

[B41] Irisarri P. Zhebentyayeva T. Errea P. Pina A. (2016). Differential expression of phenylalanine ammonia lyase (PAL) genes implies distinct roles in development of graft incompatibility symptoms in *Prunus*. *Sci. Hortic.* 204 16–24. 10.1016/j.scienta.2016.03.025

[B42] Irisarri P. Zhebentyayeva T. Errea P. Pina A. (2019). Inheritance of self- and graft-incompatibility traits in an F1 apricot progeny. *PLoS One* 14:e0216371. 10.1371/journal.pone.0216371 31071130 PMC6508642

[B43] Klagges C. Campoy J. A. Quero-García J. Guzmán A. Mansur L. Gratacós E. (2013). Construction and comparative analyses of highly dense linkage maps of two sweet cherry intra-specific progenies of commercial cultivars. *PLoS One* 8:e054743. 10.1371/journal.pone.0054743 23382953 PMC3561380

[B44] Lander E. Kruglyak L. (1995). Genetic dissection of complex traits: guidelines for interpreting and reporting linkage results. *Nat. Genet.* 11 241–247. 10.1038/ng1195-241 7581446

[B45] Li H. Durbin R. (2009). Fast and accurate short read alignment with Burrows-Wheeler transform. *Bioinformatics* 25 1754–1760. 10.1093/bioinformatics/btp324 19451168 PMC2705234

[B46] Lv W. Zheng X. Kuang Y. Cao D. Yan Y. Sun X. (2016). QTL variations for growth-related traits in eight distinct families of common carp (cyprinus carpio). *BMC Genet.* 17:65. 10.1186/s12863-016-0370-9 27150452 PMC4858896

[B47] Makhanova N. Morgan A. P. Kayashima Y. Makhanov A. Hiller S. Zhilicheva S. (2017). Genetic architecture of atherosclerosis dissected by QTL analyses in three F2 intercrosses of apolipoprotein E-null mice on C57BL6/J, DBA/2J and 129S6/SvEvTac. *PLoS One* 12:e0182882. 10.1371/journal.pone.0182882 28837567 PMC5570285

[B48] Mo Z. Feng G. Su W. Liu Z. Peng F. (2018). Transcriptomic analysis provides insights into grafting union development in pecan (*Carya illinoinensis*). *Genes* 9:71. 10.3390/genes9020071 29401757 PMC5852567

[B49] Moreno M. A. Gaudillere J. P. Moing A. (1994). Protein and amino acid content in compatible and incompatible peach/plum grafts. *J. Hort. Sci.* 69 955–962.

[B50] Muñoz-Sanz J. V. Zuriaga E. Badenes M. L. Romero C. (2017). A disulfide bond A-like oxidoreductase is a strong candidate gene for self-incompatibility in apricot (*Prunus armeniaca*) pollen. *J. Exp. Bot.* 68 5069–5078. 10.1093/jxb/erx336 29036710 PMC5853662

[B51] Musacchi S. Pagliuca G. Kindt M. Piretti M. V. Sansavini S. (2000). Flavonoids as markers for pear-quince graft incompatibility. *Angewandte Botanik* 74 206–211.

[B52] Nocker S. V. Gardiner S. E. (2014). Breeding better cultivars, faster: applications of new technologies for the rapid deployment of superior horticultural tree crops. *Hortic. Res.* 1 1–8. 10.1038/hortres.2014.22 26504538 PMC4596317

[B53] Paniagua C. Bilkova A. Jackson P. Dabravolski S. Riber W. Didi V. (2017). Dirigent proteins in plants: modulating cell wall metabolism during abiotic and biotic stress exposure. *J. Exp. Bot.* 15 3287–3301. 10.1093/jxb/erx141 28472349

[B54] Peterson G. Dong Y. Horbach C. Fu Y.-B. (2014). Genotyping-By-sequencing for plant genetic diversity analysis: a lab guide for SNP genotyping. *Diversity* 6 665–680. 10.3390/d6040665

[B55] Pina A. Cookson S. Calatayud A. Trinchera A. Errea P. (2017). “Physiological and molecular mechanisms underlying graft compatibility,” in *Vegetable Grafting: Principles and Practices*, eds Colla G. Perez-Alfocea F. Schwarz D. (Wallingford: CABI Publising), 132–154.

[B56] Prodhomme D. Valls Fonayet J. Hévin C. Franc C. Hilbert G. De Revel G. (2019). Metabolite profiling during graft union formation reveals the reprogramming of primary metabolism and the induction of stilbene synthesis at the graft interface in grapevine. *BMC Plant. Biol.* 19:599. 10.1186/s12870-019-2055-9 31888506 PMC6937855

[B57] Reig G. Salazar A. Zarrouk O. Forcada C. F. Val J. Moreno M. Á (2019). Long-term graft compatibility study of peach-almond hybrid and plum based rootstocks budded with European and Japanese plums. *Sci. Hortic.* 243 392–400. 10.1016/j.scienta.2018.08.038

[B58] Ruiz D. Lambert P. Audergon J. M. Dondini L. Tartarini S. Adami M. (2010). Identification of QTLs for fruit quality traits in apricot. *Acta Hortic.* 862 587–592. 10.17660/ActaHortic.2010.862.93

[B59] Salazar J. A. Pacheco I. Shinya P. Zapata P. Silva C. Aradhya M. (2017). Genotyping by sequencing for SNP-based linkage analysis and identification of QTLs linked to fruit quality traits in Japanese Plum (*Prunus salicina* Lindl.). *Front. Plant Sci.* 8:476. 10.3389/fpls.2017.00476 28443103 PMC5386982

[B60] Salazar J. A. Pacheco I. Silva C. Zapata P. Shynia P. Ruiz D. (2019). Development and applicability of GBS approach for genomic studies in Japanese plum (*Prunus salicina* Lindl.). *J Hortic. Sci. Biotechnol.* 94 284–294. 10.1080/14620316.2018.1543559

[B61] Salazar J. A. Ruiz D. Egea J. Martínez-Gómez P. (2013). Transmission of fruit quality traits in apricot (*Prunus armeniaca* L.) and analysis of linked quantitative trait loci (QTLs) using simple sequence repeat (SSR) markers. *Plant Mol. Biol. Rep.* 31 1506–1517. 10.1007/s11105-013-0625-9

[B62] Scheben A. Batley J. Edwards D. (2017). Genotyping-by-sequencing approaches to characterize crop genomes: choosing the right tool for the right application. *Plant Biotechnol. J.* 15 149–161. 10.1111/pbi.12645 27696619 PMC5258866

[B63] Suto J. I. Kojima M. (2019). Effects of quantitative trait loci determining testicular weight in DDD/Sgn inbred mice are strongly influenced by circulating testosterone levels. *Asian Austr. J. Anim. Sci.* 7 1826–1835. 10.5713/ajas.18.0783 31010981 PMC6819690

[B64] Truong H. T. Ramos A. M. Yalcin F. de Ruiter M. van der Poel H. J. A. (2012). Sequence-based genotyping for marker discovery and co-dominant scoring in germplasm and populations. *PLoS One* 7:e37565. 10.1371/journal.pone.0037565 22662172 PMC3360789

[B65] Usenik V. Krška B. Vičan M. Štampar F. (2006). Early detection of graft incompatibility in apricot (*Prunus armeniaca* L.) using phenol analyses. *Sci. Hortic.* 109 332–338. 10.1016/j.scienta.2006.06.011

[B66] Van Ooijen J. (2006). *JoinMap 4, Software for the Calculation of Genetic Linkage Maps in Experimental Populations.* Wageningen: Kyazma B. V.

[B67] Van Ooijen J. W. (2009). *MapQTL version 6.0, Software for The mapping of Quantitative Trait Loci in Experimental Populations of Diploid Species.* Wageningen: Kyazma B. V.

[B68] Verde I. Abbott A. G. Scalabrin S. Jung S. Shu S. Marroni F. (2013). The high-quality draft genome of peach (*Prunus persica*) identifies unique patterns of genetic diversity, domestication and genome evolution. *Nat. Genet.* 45 487–494. 10.1038/ng.2586 23525075

[B69] Verde I. Jenkins J. Dondini L. Micali S. Pagliarani G. Vendramin E. (2017). The Peach v2.0 release: high-resolution linkage mapping and deep resequencing improve chromosome-scale assembly and contiguity. *BMC Genomics* 18:225. 10.1186/s12864-017-3606 PMC5346207 28284188

[B70] Vilanova S. Romero C. Abbott A. G. Llácer G. Badenes M. L. (2003). An apricot (*Prunus armeniaca* L.) F2 progeny linkage map based on SSR and AFLP markers, mapping plum pox virus resistance and self-incompatibility traits. *Theor. Appl. Genet.* 107 239–247. 10.1007/s00122-003-1243-y 12845439

[B71] Voorrips R. E. (2002). MapChart: software for grafical presenttaion of linkage maps and QTLs. *J. Hered.* 93 73–78. 10.1093/jhered/93.1.77 12011185

[B72] Wang L. Li G. Wu X. Xu P. (2016). Comparative proteomic analyses provide novel insights into the effects of grafting wound and hetero-grafting per se on bottle gourd. *Sci. Hortic.* 200 1–6. 10.1016/j.scienta.2015.12.056

[B73] Ward J. A. Bhangoo J. Fernández-Fernández F. Moore P. Swanson J. D. Viola R. (2013). Saturated linkage map construction in *Rubus idaeus* using genotyping by sequencing and genome-independent imputation. *BMC Genomics* 14:2. 10.1186/1471-2164-14-2 23324311 PMC3575332

[B74] Wu T. D. Nacu S. (2010). Fast and SNP-tolerant detection of complex variants and splicing in short reads. *Bioinformatics* 26 873–881. 10.1093/bioinformatics/btq057 20147302 PMC2844994

[B75] Zhang J. Sun H. Yang L. Jiang F. Zhang M. Wang Y. (2019). Construction of a high-density linkage map and QTL analysis for pistil abortion in apricot (*Prunus armeniaca* L.). *Can. J. Plant. Sci.* 99 599–610. 10.1139/cjps-2018-0177

[B76] Zhebentyayeva T. Ledbetter C. Burgos L. Llácer G. (2012). “Apricot,” in *Fruit Breeding*, eds Badenes M. L. Byrne D. H. (Boston, MA: Springer), 415–458. 10.1007/978-1-4419-0763-9_12

[B77] Zhebentyayeva T. Sisco P. H. Georgi L. L. Jeffers S. N. Perkins M. T. James J. B. (2019). Dissecting resistance to *Phytophthora cinnamomi* in interspecific chestnut populations using high-throughput genotyping and QTL mapping. *Phytopathology* 109 1594–1604. 10.1094/phyto-11-18-0425-R 31287366

[B78] Zuriaga E. Soriano J. M. Zhebentyayeva T. Romero C. Dardick C. Cañizares J. (2013). Genomic analysis reveals MATH gene(s) as candidate(s) for plum pox virus (PPV) resistance in apricot (*Prunus armeniaca* L.). *Mol. Plant Pathol.* 14 663–677. 10.1111/mpp.12037 23672686 PMC6638718

