# Supplementary figures and images for: Mapping Quantitative Trait Loci Associated With Graft (In)Compatibility in Apricot (Prunus armeniaca L.)
Source: Front Plant Sci. 2021 Feb 19;12:622906. doi: 10.3389/fpls.2021.622906 (PMC7933020; doi:10.3389/fpls.2021.622906)

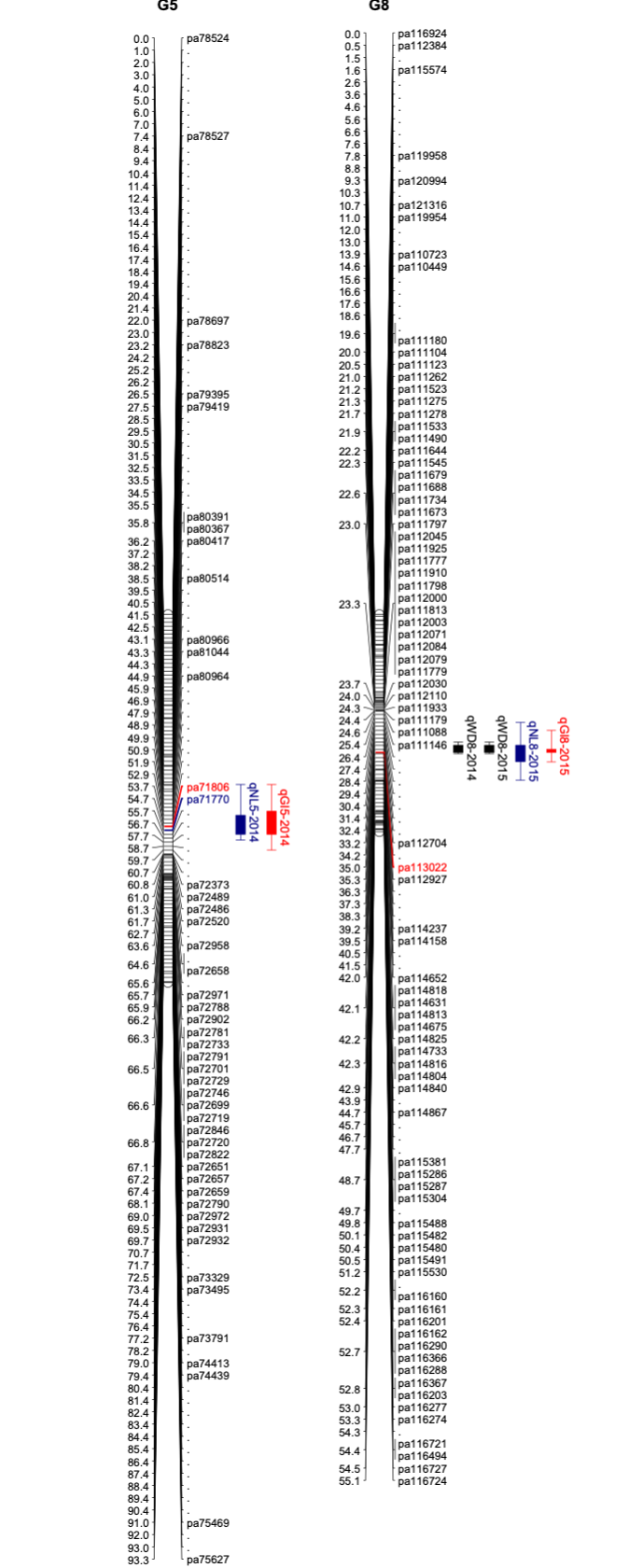

Supplement: Supplementary Figure 1 — Colocalization of quantitative trait loci (QTLs) for graft (in)compatibility traits in the “Mo × Pa” cross. QTLs for necrotic line (qNL), wood discontinuity (qWD), and overall graft (in)compatibility (qGI) detected in 2014 and 2015 were drawn as blue, black, and red bars, respectively, along linkage groups with MapChart 3.0 (Voorrips, 2002). The most significant markers associated with QTLs and their positioning on maps are shown. [file Data_Sheet_1.ZIP › Supplementary material R1/Figure S1.tiff]
